# Continental degassing of helium in an active tectonic setting (northern Italy): the role of seismicity

**DOI:** 10.1038/s41598-019-55678-7

**Published:** 2020-01-13

**Authors:** Dario Buttitta, Antonio Caracausi, Lauro Chiaraluce, Rocco Favara, Maurizio Gasparo Morticelli, Attilio Sulli

**Affiliations:** 1 Istituto Nazionale di Geofisica e Vulcanologia, sezione di Palermo, Palermo, Italy; 2 Istituto Nazionale di Geofisica e Vulcanologia, Osservatorio Nazionale Terremoti, Roma, Italy; 3 Dipartimento di Scienze della Terra e del Mare, Università di Palermo, Palermo, Italy

**Keywords:** Solid Earth sciences, Geochemistry

## Abstract

In order to investigate the variability of helium degassing in continental regions, its release from rocks and emission into the atmosphere, here we studied the degassing of volatiles in a seismically active region of northern Italy (Mw_MAX_ = 6) at the Nirano-Regnano mud volcanic system. The emitted gases in the study area are CH_4_–dominated and it is the carrier for helium (He) transfer through the crust. Carbon and He isotopes unequivocally indicate that crustal-derived fluids dominate these systems. An high-resolution 3-dimensional reconstruction of the gas reservoirs feeding the observed gas emissions at the surface permits to estimate the amount of He stored in the natural reservoirs. Our study demonstrated that the *in-situ* production of ^4^He in the crust and a long-lasting diffusion through the crust are not the main processes that rule the He degassing in the region. Furthermore, we demonstrated that micro-fracturation due to the field of stress that generates the local seismicity increases the release of He from the rocks and can sustain the excess of He in the natural reservoirs respect to the steady-state diffusive degassing. These results prove that (1) the transport of volatiles through the crust can be episodic as function of rock deformation and seismicity and (2) He can be used to highlight changes in the stress field and related earthquakes.

## Introduction

Large-scale vertical transport of fluids through the continental crust is not always dominated by steady-state diffusion processes but it can be also advective and episodic^1–4^. It has been recognized that in continental regions volatiles degassing mainly occurs in areas characterised by extensional tectonic and often coincides with seismically active zones^5^. This correspondence has been related to the enhanced permeability of the extensional tectonic discontinuities^6^. However, it has been also observed that variations of the volatiles degassing rate are earthquake-related, and are frequently post-seismic^7–9^. Thus, fluids geochemistry provides evidences of the episodic large-scale transport of volatiles. Even if the flux of the major natural gases (i.e., CO_2_) towards the atmosphere is measured at surface it is difficult to constrain the processes controlling its variability because fluids are often reactive (e.g. water-gas-rock interaction) and several processes work concomitantly during the fluids transfer through the crust^10^. Therefore, in order to figure out the relationships between fluids and earthquakes generation processes, at the base of a possible modern earthquake forecast approach, it is fundamental to unravel the processes causing and regulating the fluids flux and chemistry.

**Figure 1:**
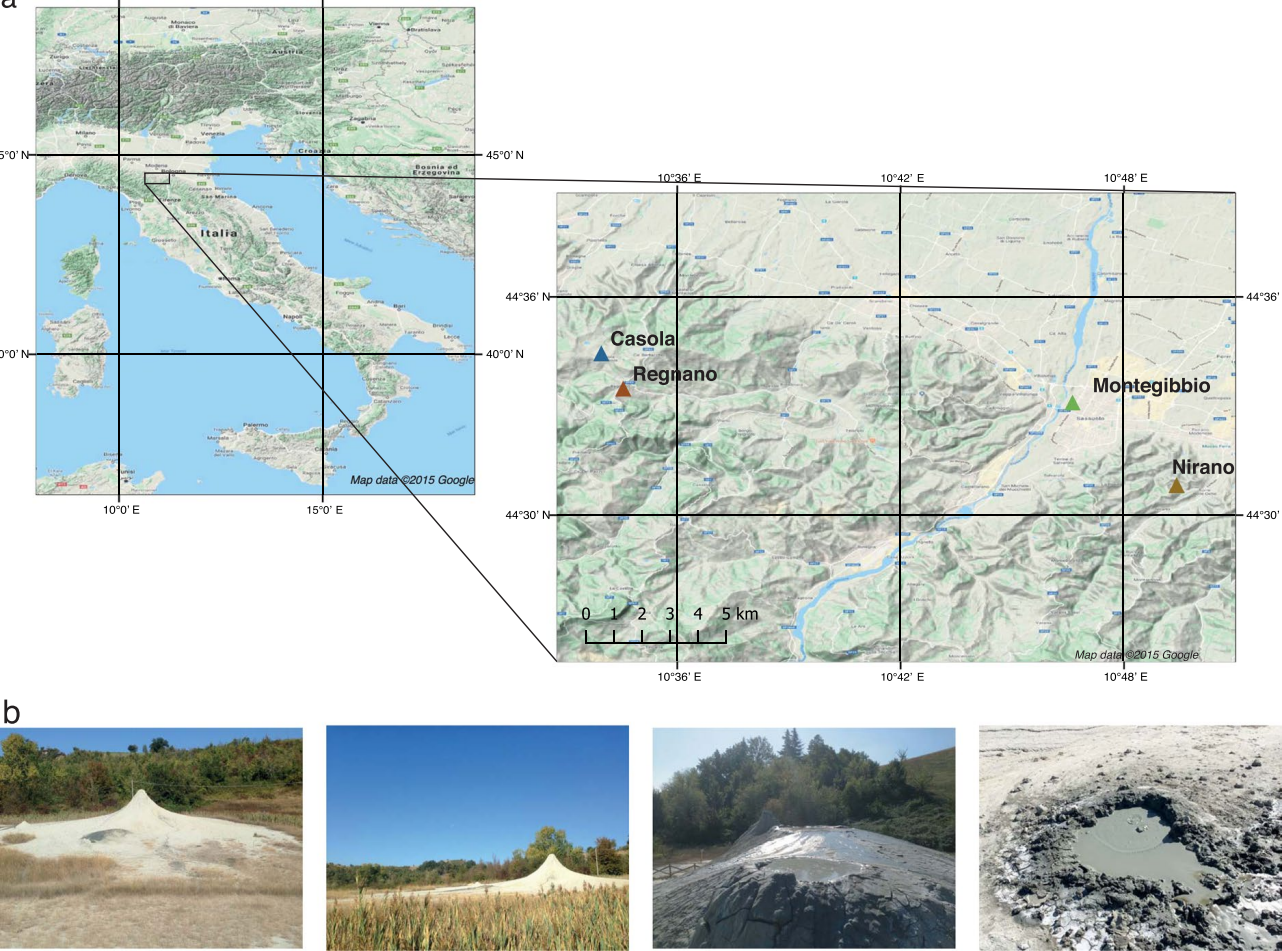
Study area. (**a**) Geographical framework with sampled sites^73^. (**b**) Some photos of Nirano and Regnano mud volcanoes during the 2018 sample campaign.

Because of their chemical properties, noble gases contribute to retrace the degassing history of the Earth and the evolution of the atmosphere^11–15^. Furthermore, for investigating the mechanism controlling the transport of fluids in continental region, He, the lightest noble gas, is largely used because its sources (air, crust and mantle) are well resolvable by the use of the isotopic ratio (^3^He/^4^He)^14^. In fact, He is characterized by two isotopes: ^3^He, which is a primordial component and it is mainly stored in the mantle, and ^4^He that is continuously produced by the U and Th decay, so its amount stored into minerals and rocks progressively increases over geological time. Melting and volatiles release from magma are the main processes that control He outgassing from the mantle. Instead, degassing of He produced in the crust occurs under different conditions mainly consisting of two stages acting at different scales: (a) the release of the volatiles from the mineral/rocks; (b) their transport towards the surface^13^. It is recognized that the crustal fluids are dominated by ^4^He, on the contrary, mantle-derived fluids show an excess of ^3^He (MORB mantle ^3^He/^4^He is 8 ± 1 Ra, where Ra is the isotopic ratio in atmosphere, 1.382 ± 0.005 × 10^−6^)^16,17^ respect to the crustal-derived fluids. He is also found in considerable quantities in some natural-gas reservoirs, where it can remain stored for millions of years^18,19^. The continental ^4^He degassing shows spatial and temporal variability at regional scale^1^, so it is an efficient tool for evaluating the role of tectonics in enhancing the crustal scale mass transport and decipherer the mechanisms of the transfer of volatiles^1,20–23^. Changes of physical properties of rocks can modify the release and transfer of volatiles through the crust^24^. For instance, rock deformation produces new (micro) fractures enhancing the release of the trapped volatiles (e.g., He) and as a consequence the fraction of volatiles released from the rocks during deformation, increases with the rock volume changes (dilatancy processes). However, even if some experimental studies show the existence of a direct link between rock deformation/fracturation and the release of He^25,26^, there are only a few of applications in natural systems^21,27,28^. In this study, we investigated the origin and processes controlling the transfer of He through the crust in a seismically active region of the Northern Apennines (Fig. 1), to establish the possible contribution of the tectonic activity in enhancing the release of volatiles accumulated in the rock over time and more specifically, how earthquakes occurrence may contribute to the episodic volatiles degassing. To this end we collect fluids from mud volcanoes (Fig. 1a,b) and we analyse their chemical and isotopic composition (He, *δ*^13^C-CO_2_, *δ*^13^C-CH_4_, *δ*^2^H-CH_4_) to figure out the origin of the outgassing volatiles and the process controlling the crustal degassing. Once verified that the volatiles comes from deep natural reservoirs, we reconstructed the reservoirs volume to estimate the total amount of He stored in these natural traps. The balance between input and output of He in the reservoirs allow us to unravel the processes that control the crustal degassing from He production until its transfer towards the surface, giving also insights to the role and modality of tectonic and seismicity in the vertical transferring of fluids (diffusive vs. advective and episodic).

## Results

In the northern Apennines (Italy) the mud volcanoes are distributed along the external sector of the fold-and-thrust belt (Fig. 1).

This region is considered a reference area to study fluid venting processes in an active fold-and-thrust belt^29^ and the fluids geochemistry is used as a potential indicator of impending local earthquakes^30^. The seismicity is concentrated in the crust and at local scale the maximum magnitude is 6.0 (https://emidius.mi.ingv.it). Furthermore, it is recognized that the fluid output from the mud volcanoes increased just after the earthquakes^9,31–33^.

**Figure 2:**
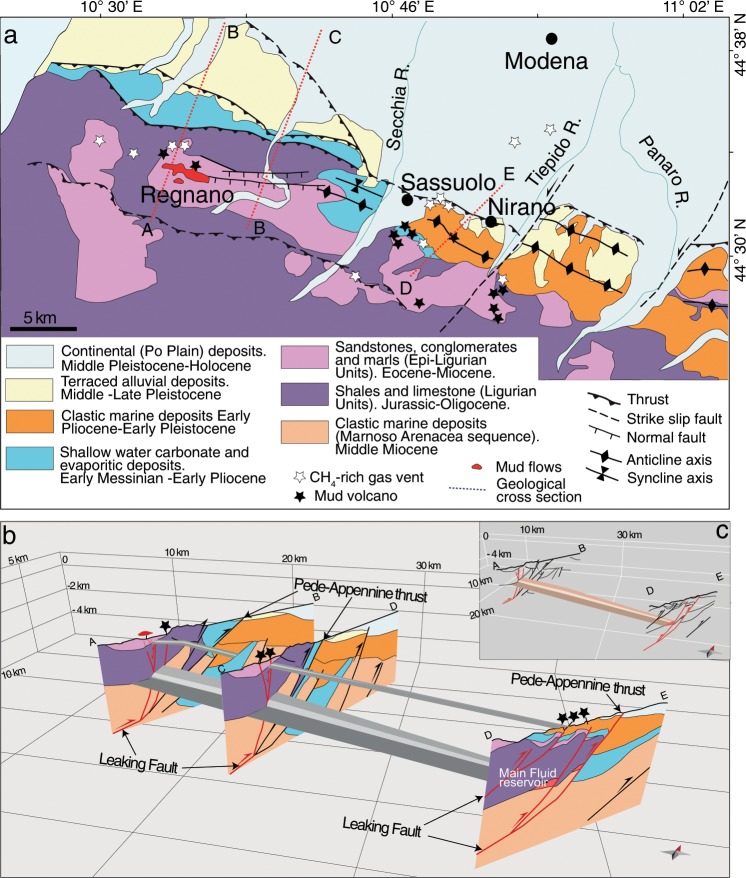
A general overview of the area with the geological units of the zone of interest (**a**) a view of the shallow and deep reservoirs by reconstruction carried out with Move 2015.1 software (**b**) deep reservoir and the system of faults that crosses it (**c**).

Previous investigations highlighted that the gases emitted from the mud volcanoes are CH_4_-rich^34^ and they are fed by two reservoirs that are strongly different in dimension and vertically separated^35^. At the regional scale, the studied mud volcanoes are localised at the top of the anticlines (Fig. 1a), particularly in joints perpendicular to the axis of the fold, where the impermeable cover allows pore fluid pressure build-up (close to lithostatic magnitudes)^35^. Mud volcanoes are placed along active normal faults (Fig. 2a), allowing a vertical migration of CH_4_ coming from deep sources (>3–6 km)^36^.

In this region mud volcanism can be potentially triggered by fault failure cycles and the overpressured fluids released during faulting^35^. Furthermore, the relatively quiescent but continuous activity of these mud volcanoes could instead reflect a short-lived leakage of overpressured fluids along permeable fractures/faults. We are therefore in the presence of a study-case site to investigate the mechanisms of fluid transfer through the crust and the possible relationships between degassing of crustal fluids and rocks deformation and seismicity.

### Fluids geochemistry

We collected gas samples from four different mud volcanoes areas (Fig. 1): Nirano, Montegibbio, Regnano and Casola. Nirano - Montegibbio sites are located 3.5 km apart, while Regnano - Casola only 2.0 km. Hereafter, we named Nirano-Montegibbio sites as the Nirano system and Regnano-Casola sites as the Regnano system. We analysed the fluids for defining the gas chemistry (He, H_2_, O_2_, N_2_, CO, CH_4_, CO_2_ and C_2_H_6_) and the isotopic composition of He (^3^He/^4^He), ^20^Ne, C of both CH_4_ and CO_2_ and H of CH_4_ (Table 1).

**Table 1:** Chemical and isotopic composition of the venting gases from the mud volcanoes of Nirano and Regnano areas.

Site	Sampling Date	Lat	Long	O_2_ (%)	N_2_ (%)	CH_4_ (%)	CO_2_ (%)	C_2_H_6_ (ppm)	He (ppm)	^20^Ne (ppm)	^4^He/^20^Ne	(R/Ra)c	Err+/−	*δ*^13^C-CO_2_	*δ*^13^C-CH_4_	*δ*^13^D-CH_4_	C(tot)/^4^He
Nirano	29/09/2018	N 44° 30′49.57″	E 10° 49′ 32.13″	0.01	0.34	98.26	0.54	381	18	0.029	636.47	0.01	1.27 × 10^−4^	+18.61	−47.2	−173	5.4 × 10^−4^
Nirano 2	29/09/2018	N 44° 30′ 46.14″	E 10° 49′ 16.90″	0.05	0.55	98.18	0.53	374	18	0.098	187.02	0.01	1.06 × 10^−4^	+13.77	−46.2	−179	5.4 × 10^−4^
Regnano	29/09/2018	N 44° 33′ 28.75″	E 10° 34′ 33.30″	0.09	0.62	97.74	1.04	1800	24	0.394	59.37	0.01	7.78 × 10^−5^	+18.41	−46.7	−170	4.2 × 10^−4^
Casola	29/09/2018	N 44° 34′ 26.56″	E 10° 33′ 57.94″	0.02	0.56	96.74	0.97	587	20	0.070	354.35	0.01	1.00 × 10^−4^	+16.08	−45.1	−176	4.8 × 10^−4^
Montegibbio	29/09/2018	N 44° 30′ 58.13″	E 10° 46′ 37.14″	bdl	01.27	97.08	0.27	135	37	0.171	215.32	0.01	9.67 × 10^−5^	n.d.	−46.2	−178	7.9 × 10^−4^

**Figure 3:**
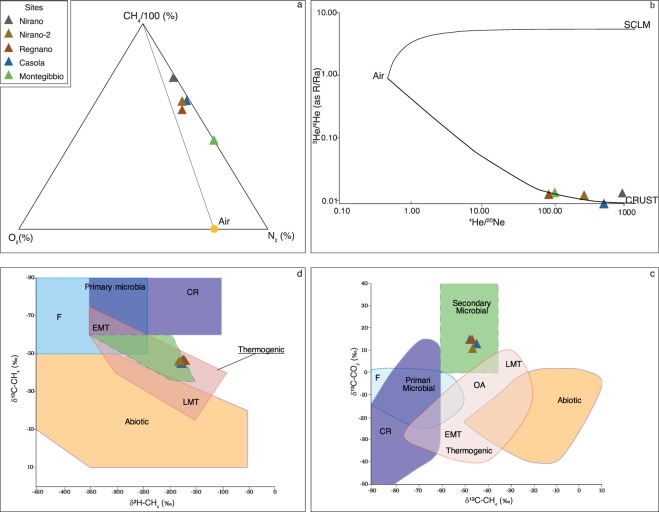
O_2_–N_2_–CH_4_/100 ternary diagram for the gas samples investigated. All the gases are CH_4_-dominated and are not along the mixing line between a pure CH_4_ end-member and air showing that the sampled gases are not air-contaminated. (**a**) A correlation diagram between the ^3^He/^4^He and ^4^He/^20^Ne ratios for the gas samples investigated. The whole black lines show the mixing lines between mantle-derived helium and between radiogenic helium with the Air. The mantle end-member is the Sub Continental Lithospheric Mantle (SCLM, 6.1 ± 0.9 Ra)^74^. (**b**) Genetic diagrams of *δ*^13^C-CH_4_ versus *δ*^13^C-CO_2_ (**c**) and methane genetic diagrams based on *δ*^2^H-CH_4_ versus *δ*^13^C-CH_4_. (**d**) T – thermogenic, A – abiogenic, CR – CO_2_ reduction, F – fermentation, OA – oil-associated thermogenic gas, LMT – late mature thermogenic gas, EMT – early mature thermogenic gas, SM-Secondary Microbial. The gaseous hydrocarbons are a mixture of secondary biogenic methane and primary and secondary thermogenic gases. The associated oils show both early and late maturities. These evidences account for different generation and migration steps, depending on burial conditions and deformation time^39^.

According to previous investigations^36–38^ the outgassing fluids from the Nirano and Regnano systems are CH_4_ dominated (96.7–98.3%). CO_2_ is up to 1.04%, O_2_ and N_2_ up to 0.09% and 1.27% respectively; He is in traces (up to 23 ppm vol.). The low concentrations of O_2_ and N_2_ indicate that the collected gases suffer low air contaminations (Fig. 3a).

The He isotopic ratios are 0.01–0.03 Ra, that is the typical range of the crustal radiogenic He (Fig. 3b). The ^4^He/^20^Ne ratios in the collected fluids are from 59.4 to 636.5 and these values are more than 2 order of magnitude higher than the same ratio in air (^4^He/^20^NeAIR = 0.318)^14^ supporting the low atmospheric component in the gases from the Nirano and Regnano systems (Fig. 3a,b). Hence, the outgassing He from the mud volcanoes systems is dominated by radiogenic ^4^He that is produced by U and Th decay in the crust^14^. The C and H isotopic composition of CH_4_ is in a good agreement with previous results highlighting the thermogenic nature of CH_4_ (Fig. 3c and Table 1)^37–39^. Here we report the first data of C and H isotopes of CH_4_ in gases from Casola and Montegibbio sites, indicating a unique origin of CH_4_ emitted from these mud volcanoes systems (Fig. 3c and Table 1). The carbon isotopic composition of CO_2_ in all the studied fluids is from +13.8‰ to +18.6‰ and according to Milkov and Etiope (2018)^40^ these values coupled to the isotopic composition of CH_4_ indicate that CO_2_ is of thermogenic origin (Table 1 and Fig. 3c,d). These results are in a good agreement with those from previous investigations^36^ that highlighted (1) the thermogenic nature of pristine methane in the deep reservoirs, (2) an origin of CH_4_ in crustal layers deeper than the reservoirs (>3–6 km) and (3) a vertical migration of CH_4_ towards the surface. Considering the main component in the collected gases is CH_4_ (~98%) and the average CH_4_/^4^He ratio in the emitted gases is ~46000 we computed the amount of ^4^He in the two reservoirs by using the total amount of gases into the reservoirs, between 4.00 × 10^11^ and 4.50 × 10^11^ standard cubic meter (SCM) for the main reservoir, and 5.68 × 10^8^ SCM for the shallow reservoir (Supplementary Information: gas reserves). So, the amount of ^4^He into the shallower reservoir and deep reservoirs are 4.90 × 10^5^ moles and 3.60 × 10^8^–4.20 × 10^8^ moles respectively. The amount of ^4^He in the shallow reservoir is three order of magnitude lower that the amount in the deep reservoir (lower than 1% of ^4^He amount in the deep reservoir), so it can be considered negligible (Table 2).

**Table 2:** Summary of reservoirs condition and initial gas in place into the traps calculated by volumetric method.

	Calculation of the initial gas in place		
	Main		Secondary
Reservoir Conditions	Trc = 327.15 °K		Trc* = 295.75 °K
	${{\rm{P}}}_{rc}^{Litho}$ = 48.98 MPa		${{\rm{P}}}_{RC}^{Litho\ast }$ = 4 MPa
G.B.V. in m^3^	9.49 × 10^9^		2.69 × 10^8^
*ϕ*	0.15		0.50*
(1-Sw-So)	0.75/0.85		0.10*
1/Bg in SCM/RCM	371.19		42.25
Z	1.1646		0.9244
	**So** = **0**.**10**	**So** = **0**	**So** = **0**
Q (SCM)	3.99 × 10^11^	4.5 × 10^11^	5.68 × 10^8^
Q (mol)	1.66 × 10^13^	1.87 × 10^13^	2.35 × 10^10^
${{\rm{Q}}}_{{}^{4}He}$ (SCM)	8.78 × 10^6^	9.94 × 10^6^	1.19 × 10^4^
${{\rm{Q}}}_{{}^{4}He}$ (mol)	3.64 × 10^8^	4.12 × 10^8^	4.93 × 10^5^

*Data from Oppo^75^.

### He degassing

There have been many consistent estimates of the flux of ^4^He from the continental crust based on calculations of *in situ* production and steady-state release to the atmosphere by using the U and Th content in rocks, crustal thickness and total release of ^4^He. These calculations yield a crustal degassing flux of ^4^He of the order 3.3 ± 0.5 × 10^10^ (atoms × m^−2^ × s^−1^)^1,15,41^. However, experimental works highlighted that the release of volatiles increases in volume of rock in an active stress field, which supports that the ^4^He degassing through the crust can be episodic in active tectonic areas^26,42,43^. Mechanical deformation and rocks failure can break (or crack) mineral grains, causing pervasive micro-fracturing and dilation. Consequently, the rocks can increase porosities from 20% to as high as 400% prior to failure^44^, open new micro-fracture surfaces, and eventually cause macroscopic failure and fracture of rocks^45^. These processes lead to a release of volatiles (e.g. He) previously trapped within mineral grains along fracture networks^46,47^ and the pore fluids transport these volatiles through the crust. Here we firstly investigate if a steady state degassing is the main process that controlled the ^4^He flux to the reservoirs below the Regnano and Nirano systems over time.

### He degassing: Steady-state conditions

The local stratigraphy and tectonic evolution indicates that the age of formation of the anticline hosting the main gas reservoirs below the Regnano and Nirano systems goes from 1.8 to 4.5 Ma. Over a million-year the flux of ^4^He through the Earth’s crust to the atmosphere is comparable to the net *in situ* production (in steady state condition), in 30–40 km of crustal thickness^48^. In order to assess if the ^4^He production in the crust and the successive migration to the natural reservoirs feeding the Nirano and Regnano systems can justify the amount of He that is stored in the reservoirs, we used a mass balance approach^49^:

1 $${}^{4}He={}^{4}H{e}_{Initial}+{}^{4}H{e}_{Insitu}+{}^{4}H{e}_{Externalflux}-{}^{4}H{e}_{Leak-d}-{}^{4}H{e}_{leak-mv}$$

where ^4^*He* represents the amount of radiogenic helium (moles) in the reservoir at time t. It includes three input and two outputs terms. Among the input terms, ^4^*He*_*Initial*_ is the amount of ^4^*He* that is in the reservoir at time t-zero, ^4^*He*_*Insitu*_ the amount of radiogenic ^4^*He* produced in the reservoir-rocks volume since its formation (from 1.8 to 4.5 Ma), ^4^*He*_*External flux*_ is the flux from the crust below the reservoirs. ^4^*He*_*Leak*−*d*_ and ^4^*He*_*Leak*−*mv*_ are the two terms of outputs: ^4^*He*_*Leak*−*d*_ is the He lost by diffusion from the main reservoir over time and it is from 4.37 × 10^5^ mol to 1.09 × 10^6^ for a t of 1.8 Ma and 4.5 Ma respectively (Methods; gas reserves). It is up to 0.36% of the total volume of He. The second one, ^4^*He*_*Leak*−*mv*_, is the He leak due to advection, and we assume that is the He emitted from the mud volcanoes. We extrapolated the current He outgassing from mud volcanoes, to the past 10^4^ years, in this time we have considered the continuous degassing (about 95 mol/y), and the possible paroxysmal activity as follows: “normal” eruption (10 times the continuous, every year) and “strong” eruption (100 times the continuous, once every 30 years)^36,50,51^. The result is that up to 3.8 × 10^7^ mol of He can by lost from mud volcanoes. Nonetheless these two outputs are less than about 10% of the amount of He stored in the main reservoir, does not entail any change in the conclusions, so it is reasonable to neglect their contribution in Eq. 1 which means an underestimation of the He present in the reservoir.

To compute ^4^*He*_*Insitu*_ and ^4^*He*_*External flux*_ we used literature data for the abundances of U and Th and for crust thickness. Hence, we based our calculations on the U and Th amounts for a Regional Refined Reference Model and the Global Refined Reference Model (Table 3)^52^.

**Table 3:** Regional and Global suite of U and Th concentration distributed in the “Sediments”, Upper Crust and Lower Crust.

	Thickness (km)	Density (g/cm^3^)	RRM-Regional*		RRM-Global*	
			U (ppm)	Th (ppm)	U (ppm)	Th (ppm)
Sediments	10	2.21	0.78 ± 0.20	1.98 ± 0.44	1.68 ± 0.2	6.91 ± 0.8
Upper Crust	10	2.80	2.20 ± 1.20	8.30 ± 4.90	2.70 ± 0.6	10.50 ± 1.0
Lower Crust	12	2.80	0.29 ± 0.24	3.17 ± 3.48	0.60 ± 0.4	3.70 ± 2.4

*Data from Coltorti *et al*.^52^.

The *in*-*situ* production of ^4^*He* is computed on the basis of the approach proposed by Zhou and Ballentine^53^:

2 $${}^{4}H{e}_{Insitu}=\rho \times \wedge \alpha \times (1-\phi )\times V\times t$$

where *ρ* is the rock density in g/cm^3^ (2.21 g/cm^3^), ∧ a parameter defining the efficiency of the transfer from the rock matrix into the gas phase, *ϕ* is the porosity of reservoir (fraction), *α* the source function of radioactive production of ^4^He in the rock matrix (mol ^4^He/grock/year), V the volume of the gas reservoir (cm^3^) and t the formation time of gas reservoirs (years). Since the process of ^4^He released from a host mineral is short compared to the geologic age^13^ ∧ can be regarded as being equal to 1. *α*, according to the U and Th decay equations, can be calculated using the following equation^54^:

3 $$\alpha =0.2355\times {10}^{-12}\times U\times [1+0.123\times (Th/U-4)]$$

where U and Th represent the concentrations of U and Th in rocks that host the reservoirs and are expressed in ppm. The accumulation rate of the *in situ* produced ^4^He of gas reservoirs is expressed as (mol/y):

4 $${q}_{{}^{4}He}^{in}=\rho \times \alpha \times (1-\phi )\times V$$

The *in*-*situ* production of ^4^He is 0.11 ± 0.02 mol/y and 0.30 ± 0.02 mol/yr (in Regional and Global model respectively).

The external flux of ^4^He is computed by using the method in Zhou and Ballentine^53^:

5 $${}^{4}H{e}_{Externalflux}={q}_{{}^{4}He}^{c}/S\times t$$

where S is the gas-bearing area of a reservoir (cm^2^), t the gas reservoir formation time (years) and ${{\rm{q}}}_{{}^{4}He}^{c}$ the average external crustal ^4^He flux (mol ^4^He/cm^2^/year). ${{\rm{q}}}_{{}^{4}He}^{c}$ can be calculated as:

6 $${q}_{{}^{4}He}^{c}=\alpha \times \rho \times H$$

where *ρ* is the average crust density in g/cm^3^ (2.80 g/cm^3^)^55^ and H is the crust thickness in cm (3.2 × 10^6^ cm, Lavecchia *et al*. 2003)^56^.

**Figure 4:**
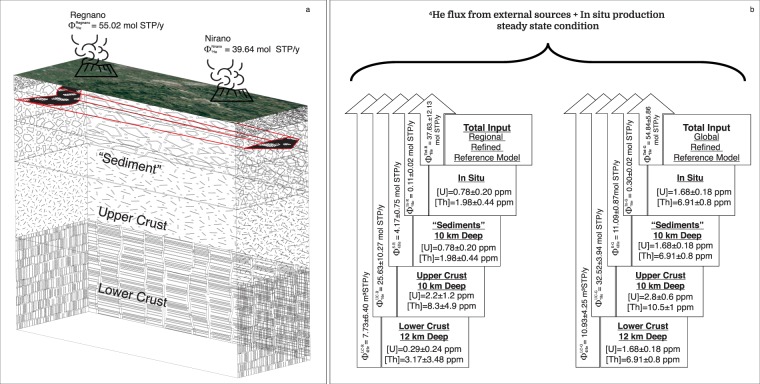
A simplified model of Nirano-Regnano mud volcanoes system. (**a**) Total input of crustal ^4^He with concentrations of Uranium and Thorium ([U] and [Th],respectively) in “Sediment”, upper and lower crust. Data of [U] and [Th] contents in rocks from Coltorti *et al*.^52^. (**b**) ${\Phi }_{{}_{4}He}^{LC-G}$ and ${\Phi }_{{}_{4}He}^{LC-R}$ are the fluxes from the lower crust with [U] and [Th] content relative to the Regional (R) and Global (G) models. ${\Phi }_{{}_{4}He}^{UC-G}$ and ${\Phi }_{{}_{4}He}^{UC-R}$ are the fluxes from the upper crust with [U] and [Th] content relative to the Regional (R) and Global (G) models. ${\Phi }_{{}_{4}He}^{S-G}$ and ${\Phi }_{{}_{4}He}^{S-R}$ are the fluxes from the “Sediment” with [U] and [Th] relative to the Regional (R) and Global (G) models. ${\Phi }_{{}_{4}He}^{In-G}$ and ${\Phi }_{{}_{4}He}^{In-R}$ are the fluxes from *in situ* production with [U] and [Th] content relative to the Regional (R) and Global (G) models. ${\Phi }_{{}_{4}He}^{Tot-G}$ and ${\Phi }_{{}_{4}He}^{Tot-R}$ are are the total input flow that feeds the reservoir with [U] and [Th] content relative to the Regional (R) and Global (G) models. ${\Phi }_{{}_{4}He}^{Nirano}$ and ${\Phi }_{{}_{4}He}^{Regnano-R}$ are the total output flow from the mud volcanoes of Nirano and Regnano.

The external flux of ^4^He to the two reservoirs is 37.53 ± 12.12 mol/y and 54.54 ± 5.86 mol/y (in Regional and Global model respectively, Fig. 4).

**Figure 5:**
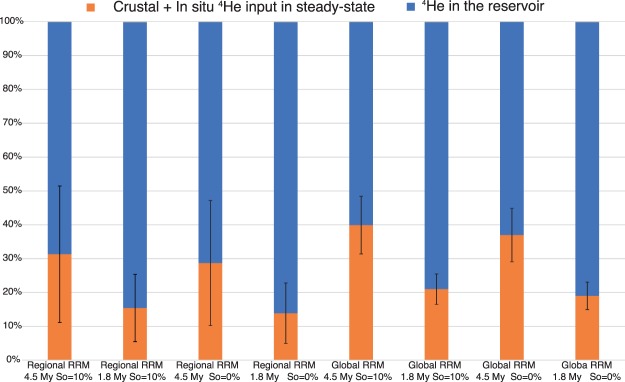
The production of radiogenic He and its release in stationary state, in the age of the trap (1.8 Ma or 4.5 Ma), represents from 15% to 40% of the helium present in the main reservoir. The error bars shown are 2*σ*.

Considering these values of ^4^He input in the reservoir, the total amount of ^4^He that can be accumulated into the trap in a time going from 1.8 and 4.5 Myr, varies from 6.7 × 10^7^ mol to 2.5 × 10^8^ mol, respectively. These values are lower than the amount of He in the reservoir that we computed by using the volumetric method (from 3.64 × 10^8^ mol to 4.12 × 10^8^ mol, Table 3). So, production of ^4^He from the whole crust below the main reservoir and its successive transfer by diffusion processes, cannot support the amount of He stored into the reservoirs (Fig. 5).

If we consider a steady-state diffusion model to explain the excess of ^4^He in the two reservoirs we need to invoke a volume of ^4^He-productive crust from 1.5 to 6.2 times larger than that below the trap (~1323 km^3^). Considering that previous investigations^38^ highlighted that the source of CH_4_ is in deep crustal layers (>3–6 km) and CH_4_ vertically migrated towards the natural reservoirs it is reasonable that processes of volatiles migration different from the steady-state diffusion occurred below the investigated mud volcanoes systems.

### ^4^He flux: Episodic degassing and active tectonic

The release of volatiles from rocks increases as effect of dilatancy and in regions affected by active tectonic, the flux of ^4^He through the crust should be higher than that in un-deforming areas where it is reasonable to assume that a diffusive steady-state transport system is acting. Our calculations show that ^4^He in the natural reservoirs is in excess respect to a steady-state whole crust degassing. As a result, in the main reservoir, there are between 1.2 × 10^8^ and 3.5 × 10^8^ moles in excess of the ^4^He produced in the crust below the main reservoir plus the ^4^*He*_*Insitu*_.

**Figure 6:**
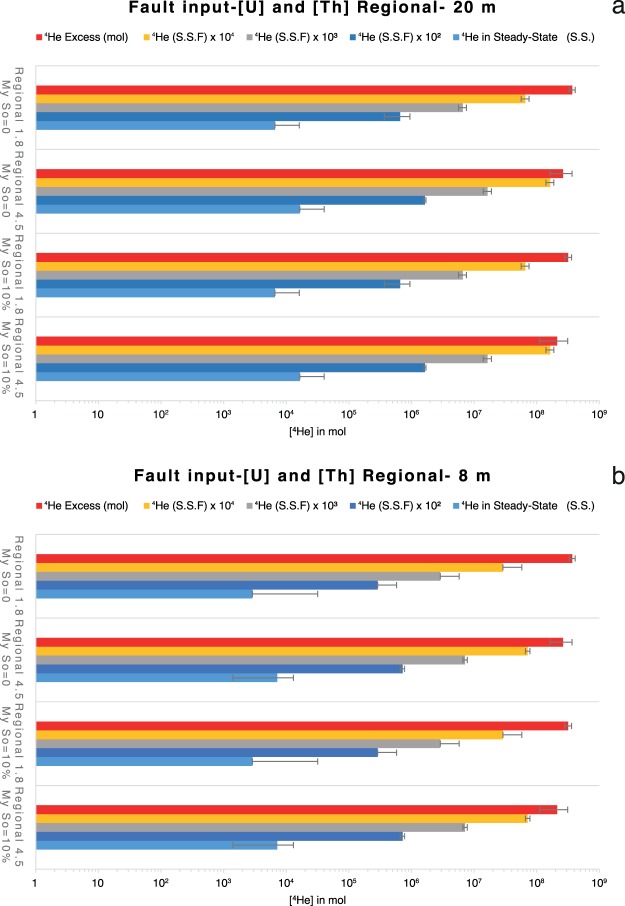
Fault contribution to the release of ^4^He. Calculated ^4^He released in Steady-state and S.S. ×10^3^–10^4^ from faults system in Nirano (lateral extension 6250 m and length of the fault zone 300 m) and Regnano (lateral extension 8750 m and length of the fault zone 500 m) for a damage zone thickness of 20 m (**a**) and 8 m (**b**) with Regional U and Th contents.

Considering that the region is tectonically active and two main systems of active faults cross cut the deep reservoir (Fig. 2c and Methods: Geological setting), here we investigate if micro-fracturing can sustain the excess of ^4^He in the reservoirs. The release of ^4^He from rock, which are affected by dilatancy, is from 10 to 10^4^ times higher than that in un-deformed rock^26^, so the release of ^4^He from a deforming fault damage zone is significantly higher if compared to the one of a tectonically undisturbed rock volume (Figs. 6 and S1).

**Figure 7:**
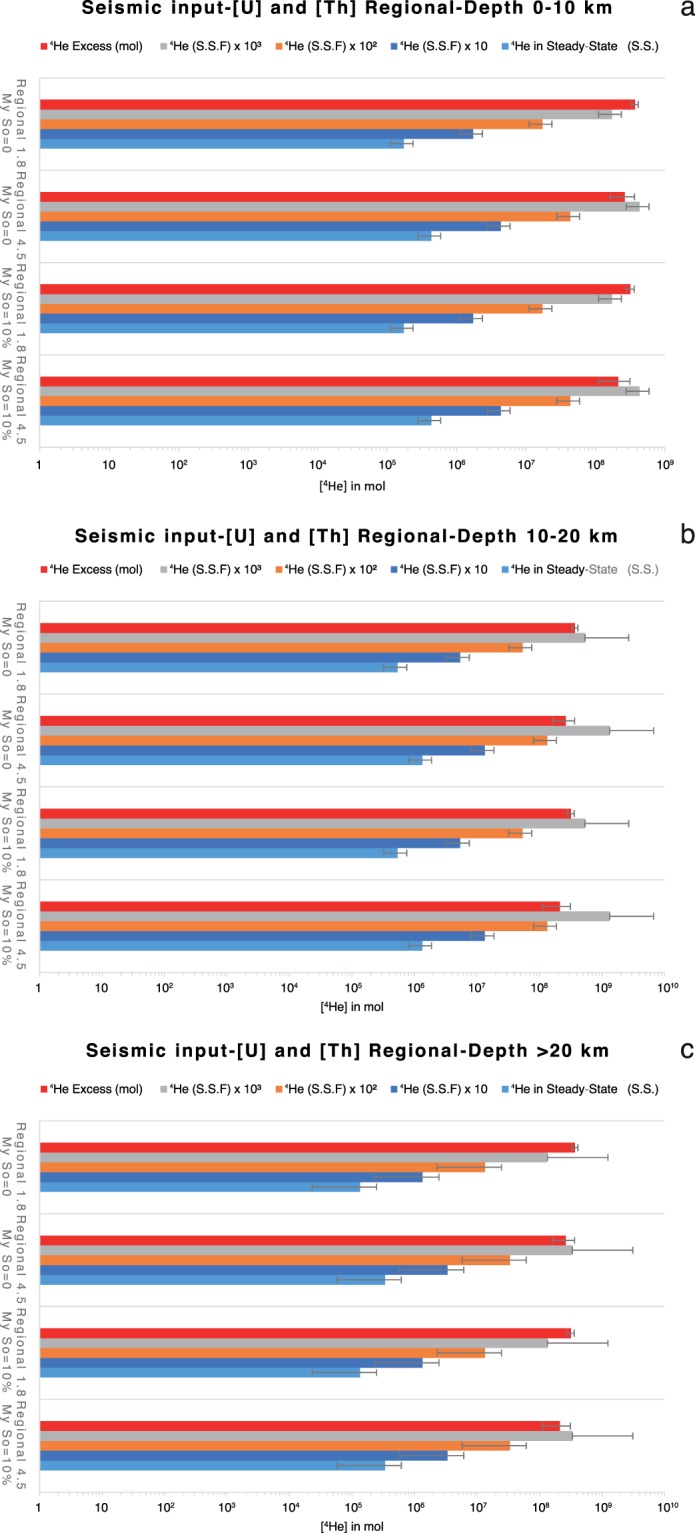
Seismic contribution to the release of ^4^He. Calculated ^4^He released in steady-state and S.S. ×10^3^–10^4^ from deformed volume of rocks by earthquakes with average annual Mw calculated by means of estimated recurrence time after frequency-magnitude distribution by Zmap7 at 0–10 km depth (**a**) 10–20 km (**b**) and 20–32 km (**c**) for the uranium and thorium contents of the Regional suite.

The volume of the damage zones of the reservoir-related faults (from 0.015 km^3^ to 0.088 km^3^; Fig. 2) releases an amount of He that matches the excess of He in the reservoirs if the release of ^4^He is 10^4^ times higher than that produces by pure diffusion process occurring within an un-deformed rock volume (Figs. 6 and S1). However, the high flux of volatiles from rocks as effect of dilatancy is not constant over time and it decreases in a scale of ka^26^. It means that in order to produce a continuous flux of volatiles high enough to justify the amount of ^4^He presumed to be into the reservoirs, the stress field has to be constantly active since the reservoir formation age. This result shows that the active regional tectonic could substantially contribute to enhance the ^4^He flux within the reservoir and that it can be an additional process to sustain the amount of ^4^He stored in the trap (Figs. 7 and S2). This implies that also the seismic activity is occurring since the origin of the gas traps.

### Seismicity and degassing

We explored the hypothesis that the excess of He in the reservoirs may be due the occurrence of local earthquakes producing micro-fractures in crustal layers. Following the approach used by Sano *et al*.^28^, which link the magnitude of an earthquake to the volume of rock affected by deformation and the related release of ^4^He, we used the local (historical and instrumental) earthquakes activity as a proxy to calculate the amount of He released by the variation of the rock volume induced by each seismic event. We used the INGV database, covering the 1986–2018 time period (http://cnt.rm.ingv.it) for the instrumental earthquakes and the historical earthquakes catalogue (https://emidius.mi.ingv.it), covering the period 1501–1997, to compute the seismic energy released by the earthquakes located below the Nirano and Regnano systems. Moreover, we also extended the catalogue to the past (in terms of geological times), by assuming the same level of seismic energy release with time, from now up to the trap formation age. The considered events occurred at depths ranging between 5 km and 31 km and in 15 km wide sector along the axis of the anticline connecting Regnano to Nirano. We firstly converted the different types of magnitude reported in the catalogues (ML and Md) in moment magnitude (Mw). For the conversion from ML to Mw, we used the coefficients proposed by Castello *et al*.^57^. While we converted the Md to Mw by using the coefficients proposed by Selvaggi *et al*.^58^. We then computed the annual average of released energy (by using ZMAP computer code) for the analysis of seismic recurrences. The annual average of released energy is about 5.48 × 10^16^ ergs/y, which corresponds to one earthquake of magnitude equal to 3.3 per year. Thus, we computed the average volume of rock deformed by the earthquakes per year^28,59^ by using the relationship:

7 $$LogV=1.06M-2.78$$

where V and M are the volume of rock affected by seismicity (km^3^) and the moment magnitude, respectively. These values correspond to a volume of deformed rock of 5.13 km^3^ per year that is higher than the volume of the faults damage zones. The amount of He released from this volume are between 7.09 × 10^−2^ and 3.58 × 10^−2^ mol/y with a Regional or Global U and Th contents in the rock respectively. So, an increase of 3 orders of magnitude of the He released by the volume of deformed rocks, due to dilatancy^26,43^, is still consistent to the amount of He estimated in the reservoirs (Figs. 7 and S2).

This result clearly indicates that the volume of deformed rock by stress field of the seismicity must be larger that the volume of the damage zone of the reservoir-related faults.

## Conclusion

Our results show that the field of stress associated to the seismicity generated a release of ^4^He from rock supporting the amount of ^4^He that accumulated in the natural reservoirs since their formation (1.8–4.5 Ma). These results demonstrate that in tectonically active regions, the crustal ^4^He degassing can episodically occur and powered as an advective process by seismo-genetic processes. In fact, in the studied area the ^4^He flux through the crust towards the atmosphere is higher than that due to a steady-state diffusive degassing and this excess can be due to the local seismicity. Considering the recognized link between rock deformation/fracturation and He degassing, the monitoring of the He flux in seismically active regions can potentially provide evidences of a modification of the field of stress due to the active tectonics, so the He can provides information to a better knowledge of the seismo-genetic processes at regional scale. However, our study shows that natural reservoirs accumulate deep sourced volatiles and the natural traps work as a sponge over time by absorbing the signal transferred towards the surface by the volatiles coming from deeper than the reservoirs, so these volatiles do not quickly reach Earth surface. These results well fit with the evidences that the increase of the activity from the mud volcanoes or vents because of earthquake is essentially post-seismic, in the sense that it occurs as a consequence of earthquakes^7,60^ and generally, no geochemical variations are recognized before. Finally, mud volcanoes are surely preferential sites for studying the relationships between fluids and seismicity, however for using He and other volatiles to investigate the genesis of earthquakes it is fundamental to have a model of fluids circulation and its storage into the crust together with an high frequency monitoring of the He that outgases at the surface. The seismogenesis is a dynamic process of ongoing rock deformation until to the fracturation, so even if fluids are directly involved in these processes nevertheless the effects of rock deformation can be also masked or reach the surface in delay.

## Methods

### Analytical procedures

Gas samples were collected in Pyrex bottles with vacuum valves at both ends, taking care to prevent air contamination, and these were analysed in 10 days from their sampling. Gas samples have been analysed in the laboratories of the Istituto Nazionale di Geofisica e Vulcanologia, sezione di Palermo. The chemical composition of He, H_2_, O_2_, N_2_, CO, CH_4_, CO_2_ and C_2_H_6_ has been measured by a Perkin Elmer Clarus 500 gas chromatograph equipped with a 3.5-m Carboxen 1000 column and double detector (hot-wire detector and flame ionization detector), with analytical errors of <3%. Analytical precision for GC analyses is better than ±5% for trace gases and ±10% for alkanes. ^3^He, ^4^He and ^20^Ne and the ^4^He/^20^Ne ratios were determined by separately admitting He and Ne into a split flight tube mass spectrometer (GVI-Helix SFT), after standard purification procedures^61^. ^3^He/^4^He ratio is expressed as R/Ra (being R the ^3^He/^4^He ratio of the sample, and Ra the ^3^He/^4^He ratio of air, 1.39 × 10^−6^).

The analytical error is generally below 0.3%. The R/Ra values were corrected for the atmospheric contamination basing on the ^4^He/^20^Ne ratio^62^ and reported as Rc/Ra (Table 1)^63^. The C isotope composition of CO_2_ (expressed as *δ*^13^C‰ vs. V-PDB) was determined using a Thermo (Finningan) Delta Plus XP CF-IRMS, connected to a Trace GC gas chromatograph and a Thermo (Finningan) GC/C III interface^63,64^. The gas chromatograph, equipped with a Poraplot-Q column (length 30 m, i.d. 0.32 mm), kept at a constant temperature of 50 °C, uses He as the carrier gas. The analytical uncertainty was ±0.1‰. Carbon and hydrogen isotopes of CH^4^ were carried out on the same equipment. GC III combustion interface was used to produce carbon dioxide from CH_4_^65^. GC-TC interface provides on-line high-temperature methane conversion into hydrogen suitable for isotope analyses. Typical reproducibility (1*σ*) for *δ*^13^C-CH_4_ and *δ*D-CH_4_ measurements is better than 0.2‰ and 2.5‰ respectively^66^.

### Gas reserves computations

The classic approach to estimate the gas reserves stored in a natural reservoir (Q) is based on a volumetric method^67^ where the computed values of the gases volume are statistically considered the “best-estimate” value^68^. Here we applied this approach to compute the total amount of gas that is stored into the deep and shallow reservoirs (Table 2).

The total gas amount in the reservoir is computed by using the equation^69^:

8 $$Q=\frac{G.B.V.\times Net/Gross\times \phi \times (1-Sw-So)}{Bg}$$

where: G.B.V. = Gross Bulk Volume represents the gross volume of mineralized rock (inclusive of any clayey and/or compact horizons that do not contribute to production) (9.5 × 10^9^ m^3^ and 2.7 × 10^8^ m^3^ the deep and the shallow gas reservoirs); Net/Gross = Ratio between the rock thickness that actually contributes to the production and the gross thickness of rock (1 for this work); $\varnothing$ = Average porosity of the reservoir (fraction, 15%); Sw = Average water saturation of the “reservoir” (fraction) o Volume fraction of porosity filled with interstitial water^70^; So = Oil saturation (between 0 and 10%); Bg = “Formation Volume Factor” that is used to express the volume of hydrocarbons originally in place at the “standard” surface conditions, i.e. at a pressure of 1 atm and at a temperature of 20 °C. The Sw it is equal to 15%, calculated with Timur 1968^71^. Where k is the permeability, equal to 80 mD.

9 $$Sw=\sqrt{\frac{{10}^{4}\times {\phi }^{4.5}}{k}}$$

The Formation Volume Factor is given by the ratio of the volume of gas to the conditions of the reservoir and the volume of the gas to the standard conditions. Mathematically:

10 $$Bg=\frac{Vrc}{Vsc}=\frac{Psc\times Trc\times Zrc}{\Pr c\times Tsc\times Zsc}$$

where, Psc, Tsc, Zsc, represent surface (pressure, temperature and compressibility factor) conditions while, Prc, Trc e Zsc, are the conditions (pressure, temperature and compressibility factor) in the reservoir. The compressibility factor, Z, is calculated by using the approach in Piper and Corredor (1993)^72^. Therefore, the reverse of the Formation Volume Factor is equal to 1/Bg = 371.19 SCM/ResCM, i.e. 1 m^3^ of pore volume under the reservoir conditions contains 1/Bg m^3^ of gas under standard conditions. Table 2 shows the total gas amount in the two reservoirs, Q: (1) from 1.66 × 10^13^ moles to 1.87 × 10^13^ moles for the deep reservoir (depth 1850–2600 m) at ${{\rm{P}}}_{rc}^{Litho}$ = 49 MPa, Trc = 327.15 °K and So from 0 to 10% and (2) 2.36 × 10^10^ moles for the shallow trap (depth 400–1000 m) at d Trc = 295.75 °K, ${{\rm{P}}}_{rc}^{Litho}$ = 4 MPa So = 0.0. The lithostatic pressure were calculated taking into account the mean thicknesses and densities of the crustal layers and the value of g.

### Helium lost by diffusion

The helium lost by diffusion it was calculated by one-dimensional steady-state diffusion model was used to quantify He loss in gas reservoirs using the formula in Liu *et al*.^49^:

11 $${\int }_{0}^{t}\frac{D}{Z}(\frac{22.4\times Q\times {C}_{(t)}}{G.B.V.\times \phi \times {K}_{{H}_{2}O}}\times \frac{{P}_{NC}\times {T}_{RC}}{{T}_{NC}}-4.5\times {10}^{-8})\times S\times dt$$

where: D is the diffusion coefficient of He (2 × 10^−6^ cm^2^/s)^49^; Z is the buried depth of reservoir, the middle point of deeper reservoir in this case (2225 m); Q is the total gas amount in the reservoir is computed by using the Eq. (8); C_(*t*)_ is the ^4^*He* concentration at time t in %v; P_*NC*_ is the normal atmosphere (1 atm); T_*NC*_ is normal temperature (273 °K); T_*RC*_ is the reservoir condition temperature (327.15 °K); G.B.V is the gross bulk volume in (9.49 × 10^11^ cm^3^); *ϕ* s the porosity (%); ${{\rm{K}}}_{{H}_{2}O}$ is the is Henry’s constant calculated from the solubility model of noble gases in the water (approximately 2500)^24^.

## Supplementary information

Supplementary Information
