# Supplementary material for: Continental degassing of helium in an active tectonic setting (northern Italy): the role of seismicity
Source: Sci Rep. 2020 Jan 13;10:162. doi: 10.1038/s41598-019-55678-7 (PMC6957705; doi:10.1038/s41598-019-55678-7)
Supplement: Supplementary file 1 — Supplementary Information [file 41598_2019_55678_MOESM1_ESM.pdf]

Continental degassing of helium in an active tectonic setting (northern Italy): the role of seismicity (supplementary informations)

Dario Buttitta[1,*], **Antonio Caracausi**[1,*], **Lauro Chiaraluce**[1], **Rocco Favara**[1], **Maurizio Gasparo Morticelli**[2], **and Attilio Sulli**[2]

[1]Istituto Nazionale di Geofisica e Vulcanologia, Italy
[2]Dipartimento di Scienze della Terra e del Mare, Università di Palermo, Palermo, Italy
[*]buttittadario@gmail.com and antonio.caracausi@ingv.it

ABSTRACT

In order to investigate the variability of helium degassing in continental regions, its release from rocks and emission into the atmosphere, here we studied the degassing of volatiles in a seismically active region of central Italy (Mw_{MAX}=6) at the Nirano-Regnano mud volcanic system. The emitted gases in the study area are CH_4–dominated and it is the carrier for helium (He) transfer through the crust. Carbon and He isotopes unequivocally indicate that crustal-derived fluids dominate these systems. An high-resolution 3-dimensional reconstruction of the gas reservoirs feeding the observed gas emissions at the surface allow us to estimate the amount of He stored in the natural reservoirs. Our study demonstrated that the in-situ production of ^{4}He in the crust and a long-lasting diffusion through the crust are not the main processes that rule the He degassing in the region. Furthermore, we demonstrated that micro-fracturation due to the field of stress that generates the local seismicity increases the release of He from the rocks and can sustain the excess of He in the natural reservoirs respect to the steady-state diffusive degassing. These results prove that 1) the transport of volatiles through the crust can be episodic as function of rock deformation and seismicity and 2) He can be used to provide highlight changes in the stress field of stress that are earthquake-related.

Geological setting

The study area is characterized by the presence of two main active mud volcanos systems (Nirano and Regnano, Figure 1 and 2 in the main text) that are located along the foothills of the Apennines on small anticlinal structures (22 km long). The presence of these structures at the top of anticlines is common because these are the structural conditions for the storage of fluids[1]. The study area develops in the external part of the NE verging Northern Apennines. It is bounded NE-wards by the Pede-Apennine thrust that separates the exposed Apennine foothills, to the southwest, from the buried chain, formed by SW dipping blind thrusts and folds, exhibiting an overall arcuate shape beneath the Po plain deposits, to the northeast. The Northern Apennines fold and thrust belt (NAFTB) formed by the deformation of the Adria continental margin since the Cretaceous. The early phase of convergence led to the subduction of the European plate below the Adria plate and the an accretionary prism resulted from the decollement of the Ligurian oceanic Units. From the Middle Eocene a change in the subduction polarity and vergence, with Adria becoming the lower plate, produced the tectonic overlapping of the accretionary prism above the Miocene siliciclastic foredeep successions (Marnoso Arenacea) deposited on the Adria plate. The thick syntectonic sedimentary wedges developed up to the Pliocene-Quaternary, showing a maximum thickness of 7–8 km. The retreating Adria subduction led to the opening of the Ligurian-Provençal and Tyrrhenian back-arc basins. The foredeep deposits were progressively incorporated into the orogenic belt, forming structural highs, which currently form the hydrocarbon traps in the subsurface of the Northern Apennines and Po Plain. Furthermore, satellite basins atop the Ligurian Units were filled during their emplacement by the Eocene-Pliocene Epiligurian successions. The Livorno-Sillaro Lineament separates the outcrop region of the Ligurian and Epiligurian units to the NW, from the Marnoso Arenacea area to the SE. The main orogenetic phase of this sector of the Northern Apennines took place between the Oligocene and early Miocene, but lasted until the Messinian. During the Quaternary, the reactivation of lateral ramps of the Miocene-Pliocene thrusts caused fluid migration and accumulation. Most of the Apennine foothills are affected by recent tectonic activity. Earthquake fault plane solutions coupled with analyses of geomorphic traces of recent faulting and deformation along the Pede-Apennine margin suggest that both frontal thrusts and lateral thrust ramps are potentially seismogenic. Along the mountain front, the activity of deep thrusts deforms the whole belt, and its

cover up to the Holocene continental deposits. Post-orogenic W-E high-angle normal faults, due to extensional stress field affecting shallow sequences, are the main pathways for the upward fluid migration and the formation of mud volcanoes at the surface, among which the Regnano and Nirano mud volcano fields, located meanly 5 km to the SW of the emergent NNE-verging Late Quaternary Pede-Apennine thrust (Fig. 1 in the main text). The Nirano mud volcano field develops in correspondence of a ramp anticline of Langhian to lower Pleistocene deposits. Geochemical analyses show that the greater fraction of the expelled fluids consists of formation water and methane generated in the Marnoso Arenacea sequence and are generally controlled by reverse faults, ramp anticline geometries, and associated fracture zones. Conversely, mud volcanism at Regnano can be related to SSW dipping normal faults developed on the hanging wall of the Pede-Apennine thrust, and rooted in the Marnoso Arenacea, which represent the main source layer of hydrocarbons. Fracture zone associated with the folding are expected to channel fluids to the Nirano field from a shallow mud reservoir located within the anticline core, below the lithological boundary between the impermeable clays (Argille Azzurre Formation) and the underlying more permeable sedimentary units, where the mud may fill the fracture network and achieve overpressuring. Instead, mud extrusion at Regnano field is directly controlled by a fault conduit funnelling the overpressured mud. Despite mud volcanism in Nirano and Regnano fields is controlled by different strain mechanisms, it can be considered as fed by a more or less continuous main reservoir settled in the anticline core developed in the hanging wall of the Pede-Apennine thrust (figure 1 in the main text). Here it is active the outgassing of volatiles methane-rich from pools and seepage in correspondence of the two mud volcanoes systems since historical times[2]. Several geological studies 1 recognized the presence of two-layered fluid reservoirs at depth feeding the gaseous emissions from the Nirano and Regnano systems and the role of the tectonic discontinuities that work as a network of pathway through which fluids mainly migrate vertically towards the surface[1].

Reservoirs and tectonic structures reconstruction

In order to evaluate the volume of the reservoirs feeding the mud volcanoes we reconstructed a 3D geological model of the area between the Regnano and Nirano mud volcanoes systems (Figure 2b in the main text), using 2D geological cross sections from previous investigations[1–3], and processed with the software Move 2015.1 (Figure 2 in the main text). After geo-referencing two NNE-SSW-oriented geological cross sections (Figure 2b) top and bottom lines of the reservoir and the faults bordering it haves been preliminarily depicted; afterwards we generated the surfaces using "create surface from line-Spline Curve method" tool. In the next step, we created the volume between the top and bottom surfaces using "Create TetraVolume Between Horizons" tool and then calculated the value. The reconstructed 3D geological model highlighted the existence of two main reservoirs (Figure 2b in the main text). A deepest one about 300-500 m thick that is located at depth-interval of 2000-2700 m of a ramp anticline with axis of 22 to 39 km, between the Regnano and Nirano areas, with a wavelength between 9 and 13 km. It is hosted into Miocene terrigenous rocks (Marnoso-Arenacea formation) characterized by 15% of porosity. The reconstructed volume is 9.49 km^3. The geological setting of the shallow reservoir, placed in the Regnano area, is more heterogeneous. It is constituted by Jurassic-Paleogene limestones pertaining to the Liguridi unit sealed upwards and laterally by Paleogene marls (Ranzano formation) with stratigraphic and tectonic contact, respectively. The fault confining laterally the shallow reservoir is the same geological structure affecting the deeper reservoir (Fig. 2 in the main text). In the Nirano sector, the shallow reservoir is made up of Miocene sands of the Epi-Liguride units sealed upwards by Plio-Pleistocene blue clays. In this case, the geological trap is the culmination of a ramp-anticline in the Ligurian units. The reconstructed volume of the shallow reservoir is 0.27 km^3. The value of volume of the shallow reservoir is one order of magnitude smaller than the deeper one, allowing us to neglect its role in the model definition. These geological traps are laterally-bordered by tectonic discontinuities, which are well recognized at depth and mapped at the surface[1]. The reconstructed geological model highlights an extensional kinematic of the fault system in the Regnano sector, where it could be interpreted as a reactivated system, while it represents a compressional fault (thrust) in the Nirano sector (Figure 2c in the main text). Moreover, they permit the transfer of fluids from the reservoirs to the surface. Brittle faults are complex volumetric zones composed of a variety of internal structures, such as slip surfaces, fault rock assemblages, and subsidiary deformation structures. The damage zone of a fault, consisting of subsidiary structures through a relatively large volume of rock surrounding the fault core, is a key factor in controlling the rock permeability and fluids flow through the crust. To investigate the rule of the tectonic discontinuities to transfer volatiles trough the crust to the reservoirs and later to the surface we computed the damage zone dimension for the faults locally cutting the upper crust. It is fundamental to know the width of these zones to assess the fluid flow towards the surface. In the absence of field structural data for the main faults (such as orientation and spatial distribution of fractures in the different fault zones)[4], the evaluation of the thicknesses of the damage-zones of the faults at local scale is obtained by using the classical evolutionary models of faulting[5–16]

and the scale-relationship between width of the damage zone and displacement. Generally, it is difficult to find a linear relationship between the damage zone width and a single parameter, therefore several parameters, such as lithology and associated diagenesis, depth of faulting, tectonic environment, and deformation mechanism, have been taken into account to evaluate its volume. To limit the effects of the different factors, we analysed previous results relatively to normal faults; moreover, we considered only two types of deformation features in the analysed rocks: deformation bands in porous sandstones and fractures in brittle rocks. In summary, the system of faults that crosses the deep reservoir consists of two main fault systems (Figure 1 in the main text). The first system mainly involves the geological domains in the area of Nirano, whose length is 300 m at a depth between 2000 and 2300 m, with a lateral extension of about 6300 m, with a possible damage zone between 8 and 20 m (measured on rocks with similar lithology)[4,17]. The second one, which affects the sector of Regnano, is 500 m long and is located at a depth between 2200 and 2700, has a lateral extension of about 8800 m and a possible damage zone between 8 and 20 m (measured on rocks with similar lithology)[4,17]

References

1. Bonini, M. Interrelations of mud volcanism, fluid venting, and thrust-anticline folding: Examples from the external northern Apennines (Emilia-Romagna, Italy). *J. Geophys. Res. Solid Earth* **112**, 1–21, DOI: 10.1029/2006JB004859 (2007).
2. Etiope, G., Martinelli, G., Caracausi, A. & Italiano, F. Methane seeps and mud volcanoes in Italy: Gas origin, fractionation and emission to the atmosphere. *Geophys. Res. Lett.* **34**, 1–5, DOI: 10.1029/2007GL030341 (2007).
3. Oppo, D., Capozzi, R. & Picotti, V. A new model of the petroleum system in the Northern Apennines, Italy. *Mar. Pet. Geol.* **48**, 57–76, DOI: 10.1016/j.marpetgeo.2013.06.005 (2013).
4. Choi, J.-H., Edwards, P., Ko, K. & Kim, Y.-S. Definition and classification of fault damage zones: A review and a new methodological approach. *Earth-Science Rev.* **152**, 70–87, DOI: https://doi.org/10.1016/j.earscirev.2015.11.006 (2016).
5. Evans, J. P. Brevia SHORT NOTES Thickness–displacement relationships for fault zones. *J. Struct. Geol.* **12**, 1061–1065 (1990).
6. Scholz, C. H., Dawers, N. H., Yu, J.-Z., Anders, M. H. & Cowie, P. A. Fault growth and fault scaling laws: Preliminary results. *J. Geophys. Res. Solid Earth* **98**, 21951–21961, DOI: 10.1029/93JB01008 (1993).
7. Savage, H. M. & Brodsky, E. E. Collateral damage: Evolution with displacement of fracture distribution and secondary fault strands in fault damage zones. *J. Geophys. Res. Solid Earth* **116**, DOI: 10.1029/2010JB007665 (2011).
8. Torabi, A. & Berg, S. S. Scaling of fault attributes: A review. *Mar. Pet. Geol.* **28**, 1444–1460, DOI: https://doi.org/10.1016/j.marpetgeo.2011.04.003 (2011).
9. Childs, C., Nicol, A., Walsh, J. J. & Watterson, J. Growth of vertically segmented normal faults. *J. Struct. Geol.* **18**, 1389–1397, DOI: https://doi.org/10.1016/S0191-8141(96)00060-0 (1996).
10. Knott, S. D. *et al.* Spatial and mechanical controls on normal fault populations. *J. Struct. Geol.* **18**, 359–372, DOI: https://doi.org/10.1016/S0191-8141(96)80056-3 (1996).
11. Vermilye, J. M. & Scholz, C. H. The process zone: A microstructural view of fault growth. *J. Geophys. Res. Solid Earth* **103**, 12223–12237, DOI: 10.1029/98JB00957 (1998).
12. Beach, A., Welbon, A. I., Brockbank, P. J. & McCallum, J. E. Reservoir damage around faults; outcrop examples from the Suez Rift. *Pet. Geosci.* **5**, 109–116, DOI: 10.1144/petgeo.5.2.109 (1999).
13. Fossen, H. & Hesthammer, J. Possible absence of small faults in the Gullfaks Field, northern North Sea: implications for downscaling of faults in some porous sandstones. *J. Struct. Geol.* **22**, 851–863, DOI: https://doi.org/10.1016/S0191-8141(00)00013-4 (2000).
14. Shipton, Z. K., Soden, A. M., Kirkpatrick, J. D., Bright, A. M. & Lunn, R. J. *How Thick is a Fault? Fault Displacement-Thickness Scaling Revisited*, 193–198 (American Geophysical Union (AGU), 2006).
15. Mitchell, T. M. & Faulkner, D. R. The nature and origin of off-fault damage surrounding strike-slip fault zones with a wide range of displacements: A field study from the Atacama fault system, northern Chile. *J. Struct. Geol.* **31**, 802–816, DOI: https://doi.org/10.1016/j.jsg.2009.05.002 (2009).

16. Faulkner, D. R., Mitchell, T. M., Jensen, E. & Cembrano, J. Scaling of fault damage zones with displacement and the implications for fault growth processes. *J. Geophys. Res. Solid Earth* **116**, DOI: 10.1029/2010JB007788 (2011).

17. McGrath, A. G. & Davison, I. Damage zone geometry around fault tips. *J. Struct. Geol.* **17**, 1011–1024, DOI: https://doi.org/10.1016/0191-8141(94)00116-H (1995).

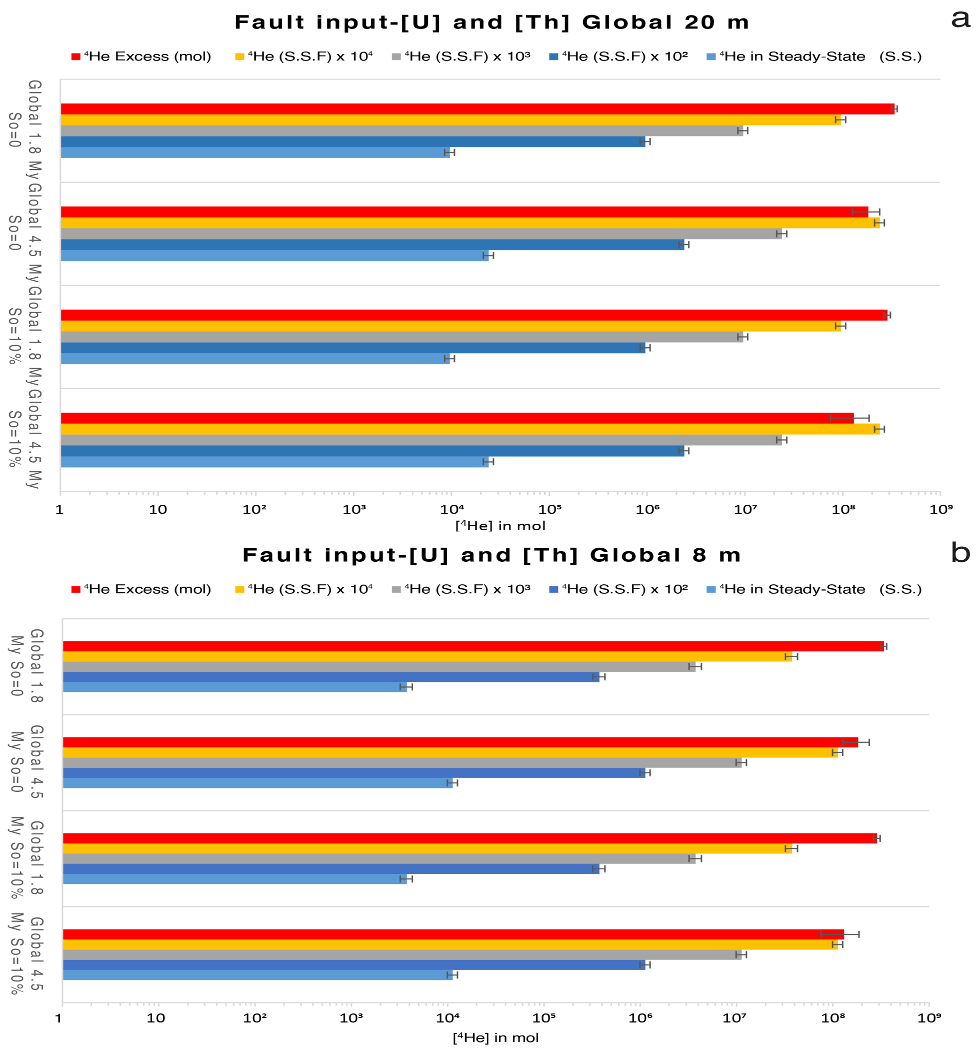

Figure S1. Fault contribution to the release of ^{4}He. Calculated ^{4}He released in Steady-state and S.S. $\times$ 10^{2}-10^{3} from faults system in Nirano (lateral extension 6250 m and length of the fault zone 300 m) and Regnano (lateral extension 8750 m and length of the fault zone 500 m) for a damage zone thickness of 20 m (a) and 8 m (b) with Global U and Th contents.

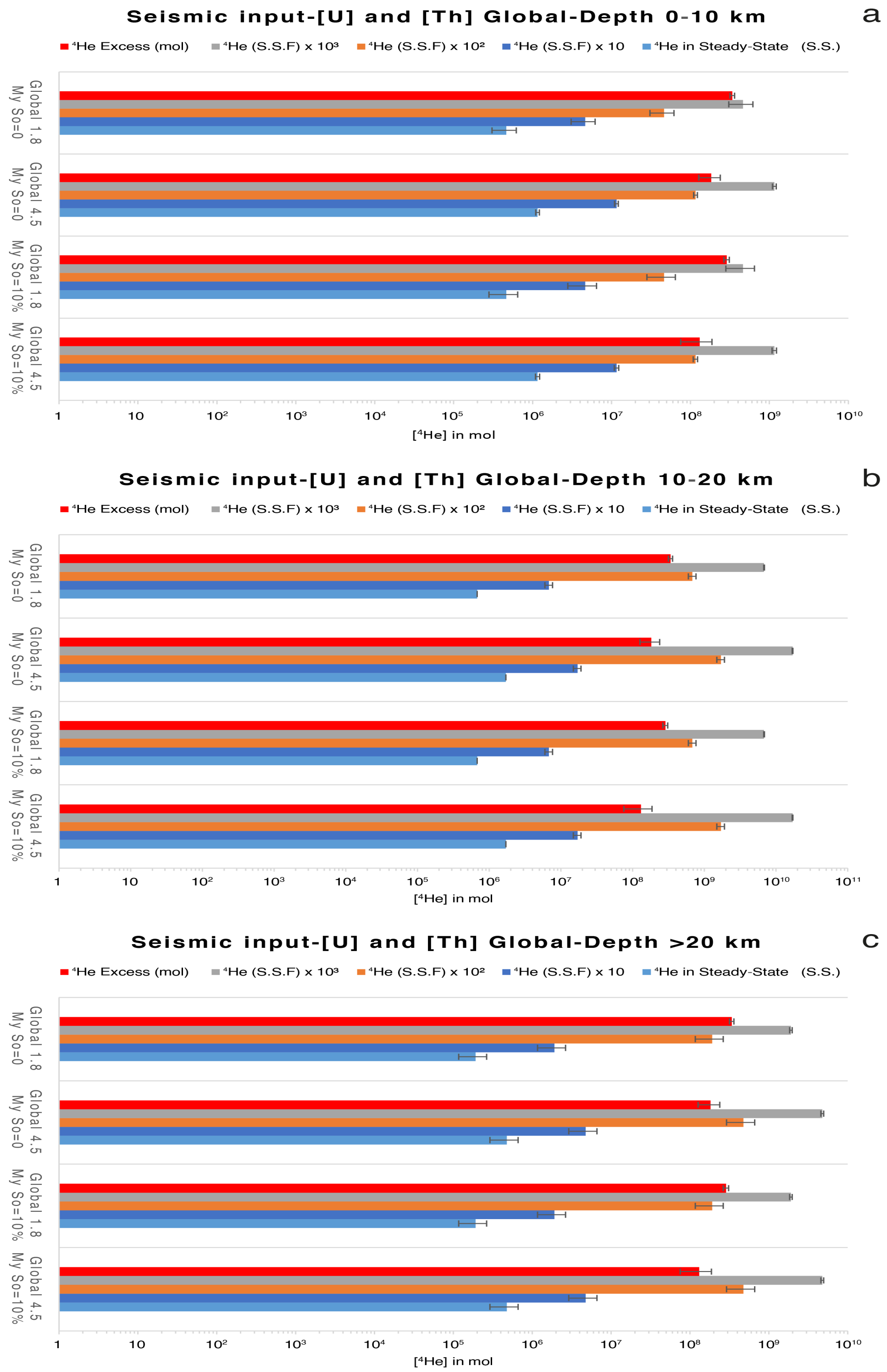

Figure S2. Seismic contribution to the release of ^{4}He. Calculated ^{4}He released in steady-state and S.S. $\times$ 10^{2}-10^{3} from deformed volume of rocks by earthquakes with average annual Mw calculated by means of estimated recurrence time after frequency-magnitude distribution by Zmap7 at 0-10 km depth (a), 10-20 km (b) and 20-32 km (c) for the uranium and thorium contents of the Global suite.
